# Sentinel Node Biopsy in Melanoma Remains a Valuable Clinical Tool. Comment on Dixon et al. Primary Cutaneous Melanoma—Management in 2024. *J. Clin. Med.* 2024, *13*, 1607

**DOI:** 10.3390/jcm14010215

**Published:** 2025-01-02

**Authors:** Thomas E. Pennington, John F. Thompson

**Affiliations:** 1 Faculty of Medicine and Health, The University of Sydney, Sydney, NSW 2050, Australia; john.thompson@melanoma.org.au; 2 Melanoma Institute Australia, The University of Sydney, Sydney, NSW 2050, Australia; 3 Department of Melanoma and Surgical Oncology, Royal Prince Alfred Hospital, Camperdown, NSW 2050, Australia

**Keywords:** melanoma, sentinel node biopsy, multidisciplinary care

## Abstract

Management of melanoma in 2024 requires at times complex decision making and a multidisciplinary approach. An article by Dixon and collaborators published in this Journal contained broad-reaching recommendations, some of which are in contradiction of accepted National and International Guidelines. This article seeks to highlight these points of contention and outline widely accepted standards of care that are considered best practice.

Proposals for the management of primary cutaneous melanoma in 2024 were outlined in a recent article in this journal [1]. However, readers should be aware that some of the proposals do not align with current practice worldwide, which is based on national and international guidelines developed after careful consideration of all available evidence. Of particular concern is the assertion that the use of sentinel node biopsy (SNB) for staging is of no value. Instead, Dixon et al. advocate for the exclusive use of six features of the primary melanoma and the patient to estimate prognosis and guide management: Breslow thickness, Age, Ulceration status, Subtype, Sex and Site. They refer to these six features together as “the BAUSSS Biomarker”. We completely agree with Dixon et al. that these are important clinicopathological features, each with prognostic significance. However, Dixon et al. pit the BAUSSS information against SNB, even though it has never been suggested that SNB should replace the features of the primary melanoma as a single prognostic factor.

SNB is widely accepted as a safe, effective and valuable tool in the management of patients with higher risk primary cutaneous melanomas, as established by several large, prospective clinical trials [2,3,4]. SNB detects nodal metastases in patients with newly-diagnosed primary melanomas at the earliest possible time, identifying those with micrometastatic disease in a SN who may benefit most from adjuvant treatment with effective modern systemic therapies which have been shown to reduce the risk of progression to stage IV disease [5]. It also has therapeutic value, greatly reducing the risk of node field recurrence.

The authors discussed relative C-statistics for the prognostic features of BAUSSS and SNB. The C-statistic is a mathematical calculation used to indicate the accuracy of a particular model or test. It gives a score of between 1.0 and 0.0, with a C-statistic of 1.0 indicating a perfect test that always predicts a certain outcome, and a value of 0.0 indicating that the test never does so. A C-statistic of 0.5 signifies a test or model that is no better at predicting an outcome than chance. Due to the heterogeneous nature of cancer and variations in biological behaviour, as well as differing outcomes of treatment, there are no tests that achieve a C-statistic of 1.0. In reality, a C-statistic of 0.70 is considered a good model and 0.80 an excellent model. Refinements to models or tests that improve the C-statistic should be welcomed.

No single feature of the primary melanoma could be considered to have the prognostic power as to predict outcome with great precision. Yet this is precisely what Dixon and colleagues suggest in their argument against the value of SNB. They combine individual factors in the BAUSSS, then pit this model against the single prognostic factor of SN status, concluding that “(SNB) is markedly inferior to BAUSS”. In fact, the addition of SN status to the important clinicopathologic features of the primary improves the C-statistic of the prognostic model by 3%, as reported by El Sharouni et al. [6]. Such an increase in a C-statistic is substantial. It would be more accurate to conclude that SNB provides prognostic precision equivalent to that of ulceration status or age, and superior precision to the other three elements of the BAUSSS system—subtype, sex and site. It has never been suggested that SNB should replace the features of the primary melanoma as a single prognostic factor.

A further, related concern is the suggestion that up-front wide excision of clinically suspected melanomas should be performed where a “broad estimate” of the Breslow thickness being below 1 mm is made, with further excision ensuing when the estimate was wrong, and the Breslow thickness was in fact > 1 mm. Dixon et al. recommend this approach “when the clinician is very confident about the clinical/dermoscopic diagnosis”.

There should be no advocacy for such an approach for several reasons: Firstly, despite the authors’ assertions to the contrary, SNB is likely to be less accurate if performed after wide excision of the primary due to potential disruption of lymphatic channels, leading to failure to identify true SNs and removal of lymph nodes that are not true SNs [7]. Secondly, it is simply not possible to reliably diagnose melanoma on clinical and dermoscopic grounds alone. All pigmented lesions carry a differential diagnosis of non-malignant and malignant mimics, and no single clinical or dermoscopic feature nor any combination of them can be considered pathognomonic. Thirdly, unnecessary wide excision of a benign lesion is clearly harmful, and should therefore be avoided, as it may increase the risk of wound complications and result in much more scarring than a simple excision-biopsy.

Dixon et al. state that the complication rate associated with SNB is 11%, without indicating the type or duration of the complications. The study quoted is a systematic review that found that the majority of complications are transient events including seroma, haematoma and infection. The pooled lymphoedema rate was 1.3% and nerve injury 0.3% [8]. When used as an argument against the use of SNB, the complication rate needs to be put into proper context, particularly when there is perceived advocacy for the use of systemic therapy without knowledge of SN status. SNB is able to identify patients at greater risk of distant relapse who may benefit from adjuvant systemic therapy. It is undeniable that a negative SNB is associated with improved prognosis across all T stages and provides patient and clinician with a more accurate estimate of mortality risk than T stage alone. Such information is valuable to inform choice, as systemic therapies can cause serious and sometimes permanent harm, maybe without benefit to the individual patient, and incur considerable cost to the patient or the health system. A balanced discussion of the relative benefits and risk of all treatment modalities in melanoma needs to consider these nuances.

The proposal by Dixon et al. that the use of SNB in older and younger age groups is unnecessary was based on seriously flawed statistical methodology, as has been discussed in detail elsewhere [9]. Suffice it to say, their argument is a philosophical one with major weaknesses, as revealed in the two theoretical examples that they gave. They firstly suggested that a 20-year-old female with a 0.4 mm ulcerated melanoma would be offered SNB based on a risk of 12% provided by the Melanoma Institute Australia Sentinel Node Metastasis Risk Calculator [10]. As members of a multidisciplinary melanoma treatment centre, we can attest that this would be a most unusual circumstance, but the predicted risk would be discussed both at a multidisciplinary team meeting and with the patient. If SNB was performed and the patient was found to be SN-positive, she would be upstaged to IIIA disease and her management and follow-up would be adjusted appropriately. Adjuvant immunotherapy might be offered in some health jurisdictions, or participation in a clinical trial. Even if young stage IIIA patients do get access to immunotherapy, preventing ten deaths from melanoma (from a theoretical cohort of 1000 patients with these clinicopathologic primary features), we would consider this entirely appropriate, not misguided, management.

At the other extreme is the authors’ argument against SNB in a cohort of 1000 eighty-year-old males with intermediate thickness melanomas, arguing that all should have immunotherapy rather than only those who were SN-positive. Using the authors asserted figures, this would expose the majority of these men (63%) to unnecessary, costly and morbid therapy, who would never gain a benefit from it. However, SNB would identify those who may benefit the most from adjuvant immunotherapy, sparing the majority from unnecessary, costly and potentially harmful systemic therapy. These two examples suggest that the authors lack an understanding of the subtleties (and practical aspects) of melanoma management in the higher risk and advanced setting, as well as what we perceive to be philosophical flaws.

As an alternative to SNB, Dixon et al. advocate use of the “Berlin” method of ultrasound with Fine Needle Biopsy (BUSFNB), asserting that in “skilled hands”, as reported by Voit et al., BUSFNB can identify early melanoma involvement in lymph nodes similar to SNB [11]. Unfortunately, the results of the BUSFNB method have not been reproduced in subsequent studies, casting doubt on its reliability [12]. Additionally, several studies have identified the size of a lymph node metastasis as an independent prognostic factor on multivariate analysis [13]. SNB carries the advantage of detection of micrometastatic deposits, which is not possible with any imaging technique. Therefore, SNB should be considered superior to nodal assessment using ultrasound or any other available imaging modality.

Dixon et al. conclude by suggesting that more trials of adjuvant drug therapy that do not require SNB are required. They claim that restricting immunotherapy drug access to those who are SN- positive alone may cement the role of SNB in the management landscape. In reality, in 2024, SNB remains the only reliable way to detect micrometastatic disease in regional lymph nodes, sparing the majority of these patients from more morbid nodal surgery that they would otherwise have required upon delayed clinical detection of metastatic nodal disease. These patients are at higher risk for distant disease relapse than patients with similar primary features who are SN-negative. Thus, SNB remains a valuable, low morbidity tool in the armamentarium of the melanoma oncologist and is entirely consistent with a modern paradigm of providing more personalised management.
